# The influence of biological sex on diagnostic markers of acute kidney injury in acute-on-chronic liver failure: insights from a single-centre tertiary care study

**DOI:** 10.1080/0886022X.2025.2553813

**Published:** 2025-09-07

**Authors:** Rohini Saha, Subhadra Priyadarshini, Shalimar, Pragyan Acharya

**Affiliations:** a Department of Biochemistry, All India Institute of Medical Sciences, New Delhi, India; b Department of Research & Development, Kalinga Institute of Medical Science (KIMS), Bhubaneswar, India; c Department of Gastroenterology, All India Institute of Medical Sciences, New Delhi, India

**Keywords:** Acute kidney injury, acute-on-chronic liver failure, sex, gender, serum creatinine, diagnostic biomarkers

## Abstract

Biological sex has a profound impact on disease severity, outcomes and diagnosis yet, its role in clinical disease is insufficiently explored. Acute on chronic liver failure (ACLF) is associated with high mortality and multiple organ dysfunctions, where acute kidney injury (AKI) significantly worsens prognosis. Here we investigated the impact of sex on the diagnostic parameters used for severity grading in ACLF. We enrolled 1,134 ACLF patients, and shortlisted 757 patients (636 males, 121 females) admitted to All India Institute of Medical Sciences, New Delhi, between 2016 and 2023. ACLF-AKI was defined and staged according to International Club of Ascites criteria. The impact of sex on baseline clinical parameters, AKI incidence, and progression were assessed using the statistical tools IBM SPSS 26.0 and GraphPad Prism 8.0. Males exhibited a higher incidence of AKI (48.34%) compared to females (28.09%). However, no significant sex-based differences were observed in AKI stages. Males also had an overall high absolute value of sCr and blood urea compared to females. However, female ACLF patients who developed AKI exhibited a significantly higher ΔsCr levels compared to males (*p* = 0.003). Kaplan-Meier analysis revealed that males developed AKI significantly faster (median 2 days) than females (median 5 days) during the first week of hospitalization. In conclusion, sex-based differences were observed in the widely used diagnostic criteria of sCr and ΔsCr for AKI in patients with ACLF. Although these findings are preliminary our results reveal sex-specific differences in sCr-based AKI diagnosis and risk stratification in ACLF which warrant further validation in prospective multi-centric cohort studies.

## Introduction

Biological sex is a key determinant of human physiology, influencing the expression of numerous genes and proteins through the presence of XX or XY sex chromosomes. While sex-based differences in disease incidence and outcomes are well recognized, their impact on diagnostic parameters remains incompletely understood.

Acute-on-chronic liver failure (ACLF) is a severe complication of cirrhosis characterized by high short-term mortality and multi-organ dysfunction [1,2]. Among these complications, acute kidney injury (AKI) markedly increases the risk of mortality [3,4]. Currently, ACLF management is largely supportive, with no targeted therapies available due to limited understanding of the underlying mechanisms of the disease [5,6]. An effective strategy for improving outcomes in ACLF would be identification of patients at risk for AKI and the implementation of nephroprotective interventions at an early stage [7].

Serum creatinine (sCr) is the primary biomarker used to diagnose AKI across etiologies, including ACLF. However, sCr levels are affected by various factors, such as diet, muscle mass, and hydration status [8]. In patients with cirrhosis, including those with ACLF, the International Club of Ascites (ICA) defines AKI based on an increase in sCr from baseline (ΔsCr), which is an improved diagnostic criteria [9]. However, the role of biological sex in determining AKI diagnostic thresholds in ACLF remains poorly characterized.

In this study, we investigated sex-based differences in AKI outcomes and diagnostic parameters in a well-characterized cohort of ACLF patients (*n* = 757).

## Methods

### Study participants

We recruited a total of 1,134 patients, admitted with ACLF diagnosis, to the Department of Gastroenterology, AIIMS, New Delhi, during the period of 2016–2023. ACLF was diagnosed and graded for organ failure using the EASL (EASL-CLIF) definition/system [10]. Patients with Grade 0 were excluded. The other exclusion criteria included presence of HCC, chronic kidney disease (CKD), diabetes, use of ATT-drugs or herbal medicines, or patients without clinical medical record.

**Figure 1.**
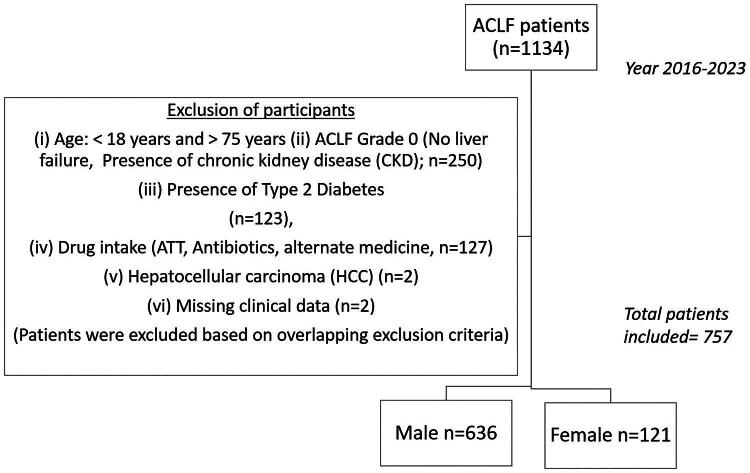
CONSORT Flow chart. A total of *n* = 1134 study participants were recruited in the study. Based on the exclusion criteria (see Supplemental Methods), *n* = 378 patients were excluded from the study. Among the included patients (*n* = 757), 83.9% patients were males (*n* = 636) and 16% patients were females (*n* = 121)

Patients were categorized by biological sex, that is males (*n* = 636) and females (*n* = 121) (Figure 1).

Acute kidney injury (AKI) and no-AKI were defined, and AKI stages and types assigned, based on the International Club of Ascites (ICA) criteria [9]. This criterion defines AKI as an increase in sCr ≥0.3 mg/dL (≥26.5 mmol/L) from baseline within 48 h or a percentage increase in sCr ≥50% from baseline. The stages of AKI were assigned as:

- Stage 1: increase in sCr ≥0.3 mg/dl (26.5 μmol/L) or an increase in sCr ≥1.5-fold to 2-fold from baseline
- Stage 2: increase in sCr >2-fold to 3-fold from baseline
- Stage 3: increase of sCr >3-fold from baseline or sCr ≥4.0 mg/dl (353.6 μmol/L) with an acute increase ≥0.3 mg/dl (26.5 μmol/L) or if the patient requires a renal replacement therapy (RRT).

However, none of the patients in our study cohort were subjected to RRT or dialysis.

Baseline refers to the most recent outpatient sCr measurement within the past 3 months. In those cases where no such measurement was available, the serum creatinine value on the day of admission (Day 0) was considered as baseline to assign AKI or no-AKI status, as described previously [10–12]. For assigning progression to AKI, patients were followed up during the hospital stay and AKI development was assessed following the ICA criteria.

The delta sCr (ΔsCr) was calculated as the difference between the creatinine levels on day 0, i.e., the day of admission, and the peak creatinine levels on subsequent days (3, 7, or 14 days), during the hospital stay. There was no discrimination in the treatment regimen in male and female patients, and standard patient management guidelines were followed uniformly.

### Statistical analysis and data representation

A *post hoc* power analysis was performed using G*Power (version 3.1.9.4) to confirm the validity of our results considering the sample size disparity between males and females. Given the observed AKI rates (48.34% in males and 28.09% in females) and sample sizes (636 males and 121 females), the study achieved 81.2% power at an alpha of 0.001. This provided adequate statistical power to detect differences in AKI incidence between the sexes.

Continuous data were presented as median with inter quartile range (Q1-Q3), while categorical data were presented as frequency and percentage. Initially, bivariate analysis was performed to assess associations, using Mann-Whitney U-test for continuous variables and Chi-square/Fisher’s exact test for categorical variables. Kaplan-Meier analysis was performed to evaluate the time-to-AKI development during the first week of hospitalization. Univariate and multivariate logistic regression was conducted to identify independent predictors of acute kidney injury (AKI). The results are reported as odds ratios (OR) with 95% confidence intervals (CI). Variables with potential significance (*p* < 0.20) in the univariate regression analysis were then included in the multivariate model to adjust for confounders. Multicollinearity among variables was checked before proceeding with the multivariate regression analysis. We performed separate sex-specific multivariable logistic regression, which avoids inappropriate comparisons across imbalanced groups and reduces the risk of overfitting. This also enabled us to evaluate sex-specific predictors of AKI. Statistical significance was set at *p* ≤ 0.05. For data visualization, Box-whiskers plots were plotted for variables with continuous data points. The median was represented with a horizontal line within each of the box, and the whiskers represent the maximum and minimum data point [13]. A multivariate binomial logistic regression was performed incorporating sex, baseline serum creatinine, serum urea, total leukocyte count (TLC), infection status, and ACLF grade to adjust for baseline severity when evaluating the independent association of sex with AKI.

Vertical histogram plots were used to plot the percentages or fractions of the total population of patients, derived from a Chi-square contingency table. The contingency table organizes data by placing one data set in rows and another in columns. The cells where the rows and columns intersect show the percentage of cases for each combination of categories [14,15], for instance AKI incidence and AKI stages. These are single-observations, hence no standard deviation or error bars were plotted. All analyses and data visualization were conducted using statistical software IBM SPSS version 26.0 and GraphPad Prism 8.0.

## Results

**Figure 2.**
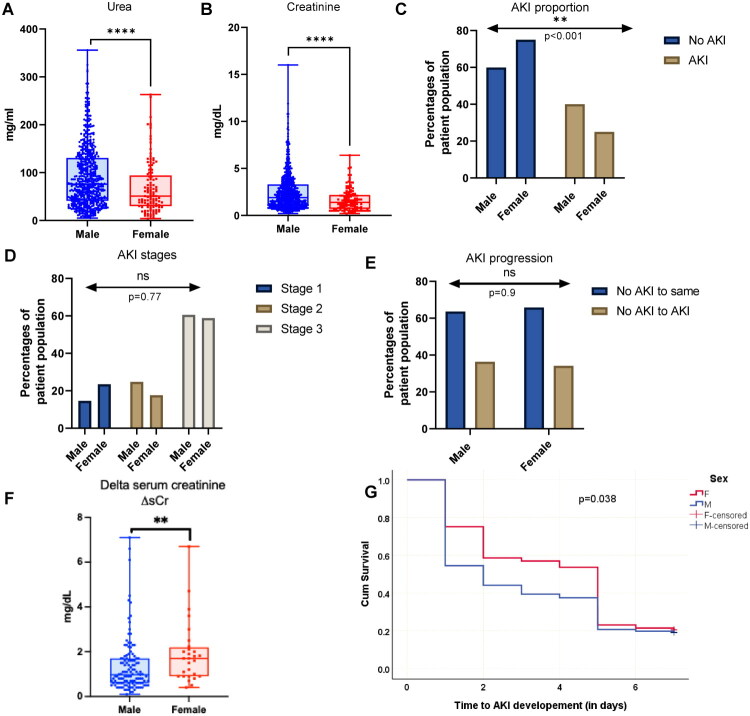
The influence of biological sex on AKI in ACLF patients. (A) Levels of blood urea (mg/mL) are significantly different in males versus females, *p* < 0.0001; (B) Levels of sCr (mg/dL) are higher in males vs females, *p* < 0.0001, (C) Males have a higher incidence of AKI than females, *p* < 0.001). (D) The distribution of stages AKI [1–3] is not influenced by sex (*p* = 0.770). (E) The percentage of ACLF no-AKI patients who progress to AKI (no-AKI to AKI) or those who do not progress (no-AKI to same) have equivalent proportion of males and females (*p* = 0.937), (F) Females (*n* = 28) who progress from no-AKI to AKI show a higher rise in sCr levels as compared to males (*n* = 123) who progress to AKI (expressed as delta serum creatinine, *p* = 0.003). (G) Kaplan Meier plot for comparing time- to- AKI development ((in days) between male and female ACLF patients during the first week of hospitalization. Females had a median AKI-free hospital stay of 5 days compared to 2 days in males. The difference was statistically significant (*p* = 0.038), suggesting earlier AKI onset in males. A, B, and F represent box-plots with median central tendency line within each box. Each dot represents individual patient values. C–E represents vertical bar plots depicts the percentage of total males or female in the cohort, derived from a Chi-squared contingency table. These percentages of patient population are single observations; hence S.D. or error bars are not plotted (See Methods).

In the overall ACLF cohort, baseline levels of serum urea and creatinine were significantly higher in males compared to age- and ACLF grade-matched females (urea: 76 mg/mL vs. 51 mg/mL; creatinine: 1.9 mg/dL vs. 1.4 mg/dL; *p* < 0.0001 for both; Figures 2A,B). To further evaluate these differences, patients were stratified by AKI status and stage according to ICA criteria [9].

A higher proportion of male patients developed AKI (48.34%) compared to females (28.09%) (Figure 2C and Table 1), though no significant differences were observed in AKI staging between the sexes (Figure 2D and Table 1). Similarly, the proportion of patients who progressed to AKI during hospitalization (within 14 days) was comparable between sexes (Figure 2E and Table 1). Among patients who progressed to AKI, the magnitude of change in serum creatinine (ΔsCr) was significantly greater in females (median ΔsCr = 1.7 mg/dL) compared to males (median ΔsCr = 0.97 mg/dL; *p* = 0.003) (Figure 2F and Table 1). However, Kaplan-Meier analysis revealed that the median time-to-AKI onset was significantly shorter in males (2 days) compared to females (5 days), (Figure 2G). This suggests a more rapid progression to AKI among male patients during early hospitalization, underscoring the need for intensified early monitoring.

Univariate analysis identified serum creatinine as the strongest predictor of AKI in both sexes, with an odds ratio (OR) of 9.018 in males (Table 2) and 14.037 in females (Table 3). In males, additional predictors included ACLF grade, presence of infection, alcohol use, serum urea, and sodium. Notably, serum globulin appeared to have a protective effect against AKI in males (OR = 0.676; Table 2). In contrast, for females, other significant variables influencing AKI included potassium, urea, and sodium (Table 3). These observations suggest sex-based differences in the underlying pathophysiology of AKI in ACLF, wherein systemic factors may play a more prominent role in males, while renal-specific parameters appear to be more predictive in females.

**Table 1.** Baseline parameters in ACLF males (*n* = 636) and females (*n* = 121) included in the study.

Parameters	Female (*n* = 121)	Male (*n* = 636)	*p*-Value
Age (years)	40 (30–50)	40 (35–48)	0.683
Haemoglobin (g/dL)	8.5 (7.2–10.4)	8.4 (6.8–10.0)	0.232
TLC (per mm^3^)	9860 (5850–16135)	13300 (8200–20000)	<0.001
Platelets (x10^3^ per mm^3^)	89.5 (59.0–142.0)	92.0 (60.0–139.0)	0.939
INR	2.700 (1.870–3.740)	2.600 (2.060–3.500)	0.771
Urea (mg/mL)	51.00 (30.00–93.50)	76.00 (41.00–130.50)	<0.0001
Creatinine (mg/dL)	1.40 (0.70–2.15)	1.90 (1.10–3.30)	<0.0001
Sodium (mEq/L)	136.0 (132.0–140.0)	136.0 (131.0–141.0)	0.358
Potassium (mEq/L)	3.90 (3.36–4.65)	4.20 (3.70–5.00)	0.003
Bilirubin (mg/dL)	15.15 (4.75–22.70)	12.70 (5.75–22.70)	0.997
Aspartate Aminotransferase (AST) (IU/L)	115.0 (58.0–216.0)	113.0 (68.0–182.0)	0.861
Alanine Aminotransferase (ALT) (IU/L)	56 (30–117)	45 (31–77)	0.181
Alkaline Phosphatase (ALP) (IU/L)	236.0 (154.0–347.0)	183.5 (130.0–271.0)	0.002
Albumin (g/dL)	2.50 (2.10–2.90)	2.50 (2.20–2.90)	0.581
Globulin (g/dL)	3.00 (2.50–3.70)	3.40 (2.70–4.10)	0.011
Presence of Infection (%)	63 (53.4%)	330 (52.9%)	0.920
AKI Proportion (%)	34 (28.09%)	307 (48.34%)	<0.001
Progression to AKI (%)	28 (34.15%)	123 (36.39%)	0.937
ΔsCr (mg/dL)	1.7 (0.9–2.20)	0.97 (0.6–1.7)	0.003
AKI Stage 1	8 (23.5%)	45 (14.65%)	0.770
AKI Stage 2	6 (17.64%)	76 (24.75%)	
AKI Stage 3	20 (58.82%)	186 (60.58%)	
Alcohol consumer (%)	8 (6.8%)	494 (79.9%)	<0.001
28-day Mortality rate (%)	75 (62.0%)	446 (70.2%)	0.072
ACLF Grade 1	12 (11.2%)	42 (7.3%)	0.524
ACLF Grade 2	27 (25.2%)	147 (25.6%)	
ACLF Grade 3	68 (63.6%)	385 (66.6%)	

Continuous data are presented as median with inter quartile range (Q1-Q3), while categorical data presented as percentage. Bivariate analysis is performed to determine p-value significance, using Mann-Whitney U-test for continuous variables and Chi-square/Fisher’s exact test for categorical variables.

**Table 2.** Univariate logistic regression analysis of factors affecting presence of AKI among males.

Male	No AKI	AKI	Univariate Logistic Regression	
			*p*-Value	Odds ratio (95% CI)
Infection (Yes)	135 (45.6%)	169 (59.7%)	< 0.001	1.768 (1.271–2.459)
Alcohol (Yes)	225 (76.5%)	231 (83.4%)	0.042	1.540 (1.016–2.334)
ACLF Grade at admission				
1	26 (9.9%)	9 (3.4%)	*Reference category*	
2	82 (31.2%)	45 (17.1%)	0.283	1.585 (0.684–3.675)
3	155 (58.9%)	209 (79.5%)	< 0.001	3.895 (1.775–8.548)
Age (years)	40 (35–47)	40 (35–49)	0.245	1.009 (0.994–1.030)
Hemoglobin (g/dL)	8.5 (6.9–10.3)	8.2 (6.6–9.8)	0.039	0.933 (0.874–0.997)
TLC (per mm^3^)	11500 (7700–18000)	15250 (8950–23565)	< 0.001	1.000 (1.000-1.000)
Platelets (x10^3^ per mm^3^)	90.0 (61.0–141.0)	92.0 (58.5–130.5)	0.372	1.001 (0.999–1.000)
INR	2.590 (2.045–3.310)	2.700 (2.070–3.600)	0.173	1.060 (0.975–1.150)
Urea (mg/mL)	47.00 (28.00–77.00)	123.00 (76.00–167.00)	< 0.001	1.027 (1.022–1.032)
Creatinine (mg/dL)	1.10 (0.80–1.70)	3.30 (2.50–4.40)	< 0.001	9.018 (6.305–12.898)
Sodium (mEq/L)	136.0 (132.0–141.0)	134.0 (130.0–140.0)	0.045	0.983 (0.966–1.000)
Potassium (mEq/L)	4.10 (3.60–4.70)	4.50 (3.70–5.30)	0.845	0.997 (0.968–1.030)
Bilirubin (mg/dL)	11.54 (5.00–20.40)	13.40 (5.80–24.00)	0.247	1.008 (0.995–1.020)
Aspartate Aminotransferase (AST) (IU/L)	106.0 (66.0–175.0)	124.0 (66.0–188.0)	0.421	1.000 (1.000-1.000)
Alanine Aminotransferase (ALT) (IU/L)	44 (30–82)	45 (31–74)	0.088	0.999 (0.998–1.000)
Alkaline Phosphatase (ALP) (IU/L)	177.5 (120.0–264.0)	181.0 (132.5–254.5)	0.689	1.000 (0.999–1.000)
Albumin (g/dL)	2.50 (2.20–2.90)	2.50 (2.10–2.80)	0.366	0.913 (0.751–1.110)
Globulin (g/dL)	3.60 (2.95–4.40)	3.30 (2.55–3.90)	< .001	0.676 (0.548–0.832)

The results are reported as odds ratios (or) with 95% confidence intervals (CI).

**Table 3.** Univariate logistic regression analysis of factors affecting presence of AKI among females.

Female	No AKI	AKI	Univariate logistic regression	
			*p*-Value	Odds ratio (95% CI)
Infection (Yes)	43 (52.4%)	19 (63.3%)	0.306	1.567 (0.663–3.701)
Alcohol (Yes)	5 (6.1%)	3 (10.0%)	0.482	1.711 (0.383–7.646)
ACLF Grade at admission				
1	9 (12.3%)	2 (7.1%)	Reference category	
2	19 (26.0%)	5 (17.9%)	0.856	1.184 (0.192–7.320)
3	45 (61.6%)	21 (75.0%)	0.369	2.100 (0.417–10.580)
Age (years)	40 (30–48)	40 (30–49)	0.407	1.014 (0.981–1.049)
Hemoglobin (g/dL)	8.7 (7.3–10.5)	8.3 (6.9–9.8)	0.549	0.945 (0.786–1.140)
TLC (per mm^3^)	9430 (5180–15201)	12100 (6600–19740)	0.075	1.000 (1.000-1.000)
Platelets (x10^3^ per mm^3^)	91.0 (60.0–147.0)	82.5 (50.0–124.0)	0.273	0.997 (0.990–1.000)
INR	2.700 (1.870–3.740)	2.900 (1.730–4.080)	0.609	1.057 (0.854–1.308)
Urea (mg/mL)	42.00 (27.00–64.00)	112.00 (85.00–130.00)	< 0.001	1.024 (1.014–1.035)
Creatinine (mg/dL)	1.10 (0.60–1.66)	2.90 (2.30–3.60)	< 0.001	14.037 (4.870–40.461)
Sodium (mEq/L)	137.0 (133.0–140.0)	134.0 (126.0–140.0)	0.044	0.946 (0.896–0.999)
Potassium (mEq/L)	3.80 (3.30–4.50)	4.30 (3.70–5.40)	0.004	1.780 (1.200–2.640)
Bilirubin (mg/dL)	15.70 (5.10–23.80)	12.75 (3.10–20.10)	0.231	0.975 (0.936–1.020)
Aspartate Aminotransferase (AST) (IU/L)	115.0 (58.0–224.0)	116.5 (56.0–167.0)	0.866	1.000 (0.998–1.002)
Alanine Aminotransferase (ALT) (IU/L)	47 (30–122)	56 (24–98)	0.658	1.001 (0.998–1.003)
Alkaline Phosphatase (ALP) (IU/L)	233.0 (133.0–337.0)	236.0 (159.0–332.0)	0.977	1.000 (0.998–1.002)
Albumin (g/dL)	2.50 (2.20–2.95)	2.55 (2.10–2.80)	0.458	0.764 (0.374–1.560)
Globulin (g/dL)	3.10 (2.55–3.80)	3.00 (2.35–3.55)	0.580	0.861 (0.507–1.460)

The results are reported as odds ratios (or) with 95% confidence intervals (CI).

Multivariate analysis of males and females performed separately confirmed serum creatinine as the most robust independent predictor of AKI in both males (OR = 9.144) and females (OR = 13.516); *p* < 0.001 for both (Table 4), as expected. While several other variables showed significance in univariate analysis among males, including infection, alcohol use, ACLF grade, and urea, these did not retain independent significance after adjustment, suggesting their effects may be mediated through interconnected clinical pathways.

**Table 4.** Multivariate logistic regression analysis of factors affecting presence of AKI in males and females as independent groups.

Predictor	Estimate	SE	*Z*	*p*-Value	Odds ratio (95% CI)
**Males**					
Intercept	−2.907	3.080	−0.944	0.345	0.055 (0.000–22.840)
Infection (Yes)	0.132	0.358	0.368	0.713	1.141 (0.566–2.300)
Alcohol (Yes)	0.117	0.443	0.265	0.791	1.124 (0.472–2.680)
ACLF Grade at admission:					
2	−0.130	0.907	−0.144	0.886	0.878 (0.148–5.200)
3	−0.130	0.874	−0.149	0.882	0.878 (0.158–4.870)
Hemoglobin (g/dL)	−0.081	0.079	−1.033	0.302	0.922 (0.790–1.080)
Urea (mg/mL)	0.005	0.005	1.135	0.256	1.005 (0.996–1.010)
Creatinine (mg/dL)	2.213	0.307	7.218	< 0.001	9.144 (5.013–16.680)
Sodium (mEq/L)	−0.002	0.019	−0.088	0.930	0.998 (0.961–1.040)
Globulin (g/dL)	−0.346	0.187	−1.852	0.064	0.707 (0.490–1.020)
**Females**					
Intercept	0.231	5.921	0.039	0.969	1.260 (0.000–138056.300)
Urea (mg/mL)	0.002	0.009	0.199	0.842	1.002 (0.985–1.020)
Creatinine (mg/dL)	2.604	0.613	4.248	< 0.001	13.516 (4.065–44.940)
Sodium (mEq/L)	−0.052	0.042	−1.216	0.224	0.950 (0.874–1.030)
Potassium (mEq/L)	0.160	0.291	0.551	0.582	1.174 (0.664–2.070)

These findings underscore the importance of recognizing sex-associated variations in AKI diagnostic parameters and highlight the need for validation through larger, multicenter prospective cohort studies to refine and personalize diagnostic approaches.

OR: Odds Ratio; CI: Confidence Interval

**Table 5.** Multivariable logistic regression model assessing independent predictors of AKI including sex and disease severity markers.

Predictor	Estimate	SE	*Z*	*p*-Value	Odds Ratio (95% CI)
Intercept	−3.699	0.60	−6.61	< 0.001	0.025 (0.008–0.074)
Sex (M vs F)	0.586	0.297	1.971	0.049	1.796 (1.003–3.216)
Creatinine (mg/dL)	0.237	0.10	2.379	0.017	1.267 (1.043–1.541)
Urea (mg/mL)	0.024	0.003	9.739	< 0.001	1.025 (1.020–1.030)
TLC (per mm^3^)	8.21e-6	1.06e-5	0.775	0.438	1.000 (0.999–1.00)
Infection	0.174	0.217	0.803	0.422	1.190 (0.773–1.82)
ACLF Grade 2 vs 1	0.107	0.525	0.204	0.838	1.113 (0.398–3.114)
ACLF Grade 3 vs 1	0.479	0.510	0.957	0.339	1.615 (0.605–4.311)

To examine whether sex remained an independent predictor of AKI after adjusting for disease severity, we performed multivariate logistic regression including baseline creatinine, serum urea, TLC, infection status, and ACLF grade at admission. Male sex was independently associated with higher odds of AKI (OR = 1.796, 95% CI: 1.003–3.216, *p* = 0.049) than female. Baseline creatinine (OR = 1.267, *p* = 0.017) and serum urea (OR = 1.025, *p* < 0.001) were also significant predictors. Neither ACLF grade, infection status, nor TLC remained significant after adjustment (Table 5).

## Discussion

In our study, we observed that within an age and ACLF-grade-matched cohort of male and female patients, kidney function parameters, specifically serum urea and creatinine, were significantly higher in males compared to females. This baseline disparity suggests sex-related physiological influences on renal biomarker levels. This difference was likely attributable to a greater incidence of ACLF-associated AKI in male patients (Figure 2A–C and Table 1). However, the severity of AKI, as assessed by AKI staging, did not differ significantly between males and females with ACLF-AKI (Figure 2D and Table 1). Similarly, the proportion of patients without AKI at admission who progressed to AKI during hospitalization was comparable across the sexes (Figure 2E and Table 1). Females who developed AKI, exhibited a steeper increase in serum creatinine (ΔsCr) compared to their male counterparts (Figure 2F and Table 1). These observations suggest that biological sex may influence both baseline renal function parameters and the subsequent trajectory of biomarker changes during AKI, which may in turn affect the interpretation and performance of diagnostic markers for AKI.

Further analysis revealed distinct sex-specific risk factors for AKI in ACLF patients. In males, serum creatinine, ACLF grade, presence of infection, alcohol use, urea, sodium, and globulin emerged as independent predictors of AKI (Table 2). In contrast, for females, serum creatinine, followed by potassium, sodium, and urea were identified as independent predictors (Table 3). Despite these differences, multivariate analysis revealed that serum creatinine was the only consistent and independent predictor of AKI in both sexes (Table 4), as expected. This reinforced the central role of creatinine in AKI diagnosis, while also suggesting that ancillary predictors may differ by sex.

A trend toward a steeper rise in serum creatinine (ΔsCr) among females following AKI onset was observed in our cohort. While exploratory, this finding raises the possibility that the performance of current AKI diagnostic thresholds may differ by sex, meriting investigation in future studies. Sex-related differences in AKI risk and progression have been documented in other clinical contexts, with males often demonstrating higher susceptibility and worse outcomes, likely influenced by hormonal, immune, and hemodynamic factors [16,17]. Put together, these findings highlight critical sex-based differences in diagnostic markers, particularly serum creatinine and ΔsCr, in ACLF patients and suggested potential avenues for deeper investigation. A previous report suggests that in hospitalized cirrhotic patients with infection, females had lower baseline and peak creatinine, similar to our study, but comparable delta creatinine and were more likely to receive RRT at equivalent creatinine levels [18]. Therefore, future studies should aim at elucidating the biological mechanisms underpinning these disparities, such as the modulatory roles of sex hormones like estrogen and testosterone, and the genetic influence of XX versus XY chromosomal composition on renal physiology and dysfunction. Immunological differences, including variations in cytokine profiles and immune cell activation between males and females, may also contribute to distinct responses in ACLF-AKI and warrant further exploration.

Although males presented with higher baseline levels of serum creatinine, urea, and leukocyte counts, our adjusted multivariate analysis identified sex as an independent factor associated with AKI diagnosis. Notably, clinical severity indicators, including AKI stage distribution, ACLF grade, and 28-day mortality, were comparable between males and females (Tables 4 and 5). This suggests that the higher incidence of AKI in males is not solely attributable to more severe disease at presentation but may also reflect a sex-related bias in creatinine-based diagnostic criteria. Our analysis further demonstrated that the male sex remained significantly associated with increased odds of AKI even after adjusting for key clinical variables such as baseline serum creatinine, urea, TLC, infection status, and ACLF grade (Table 5). This supports the hypothesis that biological sex may exert an independent influence on AKI risk, beyond traditional severity markers. Although the effect size was modest (adjusted OR ∼1.8), the finding raises the possibility of underlying biological or physiological mechanisms that increase AKI vulnerability in males with ACLF. Further mechanistic investigations and prospective validation studies are needed to better understand and substantiate this association.

Our Kaplan-Meier time-to-event analysis demonstrated that male patients developed AKI significantly earlier during hospitalization than females (median 2 vs. 5 days, respectively; log-rank *p* ≤ 0.05; Figure 2G). This time disparity suggests potential biological or physiological differences in the AKI trajectory between the sexes in the context of ACLF. One possible explanation is that males, who had higher baseline serum creatinine levels, may cross the diagnostic threshold for AKI more rapidly even with modest renal insults. Sex-based differences in renal hemodynamics, tubular function, or systemic inflammation may influence susceptibility to early renal deterioration [19–26]. Estrogen has been reported to confer nephroprotection in various contexts, including ischemic and septic AKI, potentially delaying the onset of detectable renal dysfunction in females. These findings have both diagnostic and therapeutic implications. The shorter time-to-AKI onset in males may narrow the window for clinical intervention, underscoring the importance of intensified early monitoring in male ACLF patients. Conversely, the delayed onset in females may provide a broader therapeutic window but could also mask injury progression under current diagnostic thresholds. While this observation requires extensive prospective validation, it supports a growing recognition that sex-based biological differences may influence both the onset and detection of AKI.

Large cohort analyses and meta-analyses have found that male patients are more susceptible to AKI, including dialysis-requiring AKI, compared to females. Hsu et al. [19] and Xue et al. [20] showed that males had a higher incidence of dialysis-requiring AKI and ARF, respectively, in large hospitalized populations. Neugarten, Golestaneh et al. [21–23] found that female sex was protective in both hospital-acquired AKI and CKD progression, with estrogen being a key factor. The ADQI 33rd consensus [24] and other recent studies [25,26] further emphasized the role of hormonal influence and age, noting that renal protection in females may diminish post-menopause (Supplementary Table 1). In contrast, a single cohort study from China did not identify any sex-related difference in the incidence of AKI among ACLF patients, highlighting the importance of geographic and cohort-specific factors, and underscoring the need for larger, well-powered studies to clarify the association between sex and AKI in this population [27].

The predominance of male patients in our ACLF cohort (84%) is consistent with previous studies that have reported the proportion of males in ACLF patients ranging from 63.3% to 90.7% across various ACLF and ACLF-AKI cohorts [10,28–31]. This male bias has been commonly observed in HBV-associated ACLF and decompensated cirrhosis cohorts as well [32,33]. This male-predominance across ACLF studies indicates a consistent demographic pattern and reinforces the external validity of our observations (Supplementary Table 2).

Overall, these findings highlight the importance of recognizing sex-associated variations in AKI diagnostic parameters and emphasize the need for validation through larger, multicenter prospective cohort studies to refine and personalize diagnostic approaches.

## Limitations

A limitation of the study was the relatively smaller number of female participants compared to males in our cohort of ACLF patients. While this distribution limits statistical power for sex-stratified analyses, it is consistent with real-world ACLF epidemiology, where male predominance has been widely reported [10,28–33]. Another limitation of our study was the inclusion of participants from a single hospital, and lack of multi-centric validation. The inclusion of a larger and more balanced cohort of male and female patients would provide greater statistical power, helping to validate these findings further.

## Supplementary Material

Supplementary Table 2 Higher male proportion in study cohorts_rev.docx

Supplementary Table 1_Gender influence in AKI.docx

## Data Availability

All data associated with this manuscript are available within the manuscript files.

## Study approval

The study was approved by the All India Institute of Medical Sciences, New Delhi ethics committee [Approval reference no. IEC.473/07.10.2016 and IEC.369/01.08.2016/RP-20/2016] and abides by the Declaration of Helsinki. Informed and written consent was obtained from each study participant and any identifiers were withheld from the study analyses.
