# Supplementary material for: The influence of biological sex on diagnostic markers of acute kidney injury in acute-on-chronic liver failure: insights from a single-centre tertiary care study
Source: Ren Fail. 2025 Sep 7;47(1):2553813. doi: 10.1080/0886022X.2025.2553813 (PMC12418795; doi:10.1080/0886022X.2025.2553813)
Supplement: Supplementary Table 2 Higher male proportion in study cohorts_rev.docx [file IRNF_A_2553813_SM2630.docx]

| **Sl. No.** | **Male (%)** | **Study groups** | **Reference** |
| --- | --- | --- | --- |
| 1. | 63.3% | ACLF and CLD (multiple etiology) | Moreau, Richard, et al. "Acute-on-chronic liver failure is a distinct syndrome that develops in patients with acute decompensation of cirrhosis." *Gastroenterology* 144.7 (2013): 1426-1437. |
| 2. | 71.4% | HBV related ACLF | Wan, Zhi-Hong, et al. "Cystatin C is a biomarker for predicting acute kidney injury in patients with acute-on-chronic liver failure." *World Journal of Gastroenterology: WJG* 19.48 (2013): 9432. |
| 3. | 70.2% | ACLF-AKI | Jha, Praveen, et al. "Baseline serum cystatin C as a marker of acute kidney injury in patients with acute-on-chronic liver failure." *Indian Journal of Gastroenterology* 40.6 (2021): 563-571. |
| 4. | 90.3% | HBV-AKI | Wan, Z., Wu, Y., Yi, J., You, S., Liu, H., Sun, Z., ... & Xin, S. (2015). Combining serum cystatin C with total bilirubin improves short-term mortality prediction in patients with HBV-related acute-on-chronic liver failure. *PloS one*, *10*(1), e0116968. |
| 5. | 90.7% | ACLF w and w/o AKI | Jiang, Q. Q., Han, M. F., Ma, K., Chen, G., Wan, X. Y., Kilonzo, S. B., ... & Ning, Q. (2018). Acute kidney injury in acute-on-chronic liver failure is different from in decompensated cirrhosis. *World Journal of Gastroenterology*, *24*(21), 2300. |
| 6. | 69% | Cirrhosis with AKI | Belcher, J. M., Sanyal, A. J., Peixoto, A. J., Perazella, M. A., Lim, J., Thiessen‐Philbrook, H., ... & TRIBE‐AKI Consortium. (2014). Kidney biomarkers and differential diagnosis of patients with cirrhosis and acute kidney injury. *Hepatology*, *60*(2), 622-632. |
| 7. | 78% | Decompensated cirrhosis | Ariza, X., Sola, E., Elia, C., Barreto, R., Moreira, R., Morales-Ruiz, M., ... & Ginès, P. (2015). Analysis of a urinary biomarker panel for clinical outcomes assessment in cirrhosis. PloS one, 10(6), e0128145. |
| 8. | 87.3% | ACLF (multiple etiology) | Sharan, K., Sharma, A., Rana, S., Patnaik, I., & Gupta, R. (2024). Neutrophil Gelatinase–associated Lipocalin Predicts Short-term Outcomes in Decompensated Cirrhosis With Acute Kidney Injury. *Journal of Clinical and Experimental Hepatology*, *14*(1), 101274. |

**Supplementary Table 2: Studies showing higher proportion of males in liver cirrhosis and ACLF patient cohorts**
