# Supplementary material for: The influence of biological sex on diagnostic markers of acute kidney injury in acute-on-chronic liver failure: insights from a single-centre tertiary care study
Source: Ren Fail. 2025 Sep 7;47(1):2553813. doi: 10.1080/0886022X.2025.2553813 (PMC12418795; doi:10.1080/0886022X.2025.2553813)
Supplement: Supplementary Table 1_Gender influence in AKI.docx [file IRNF_A_2553813_SM2629.docx]

**Supplementary Table 1. Literature review on influence of gender in AKI**

| **Sl. No.** | **Finding on gender Bias** | **Study groups** | **Reference** |
| --- | --- | --- | --- |
| 1 | Males had higher risk of acute renal failure (ARF) | *“Medicare”* beneficiaries cohort patients | Xue, Jay L., et al. "Incidence and mortality of acute renal failure in Medicare beneficiaries, 1992 to 2001." *Journal of the American Society of Nephrology* 17.4 (2006): 1135-1142. |
| 2 | Males had higher risk of dialysis-requiring AKI | General hospitalized population | Hsu, Raymond K., et al. "Temporal changes in incidence of dialysis-requiring AKI." *Journal of the American Society of Nephrology* 24.1 (2013): 37-42. |
| 3 | Female sex confers renal protective role in AKI development | Meta-analysis of 83 patient cohorts | Neugarten, Joel, and Ladan Golestaneh. "Female sex reduces the risk of hospital-associated acute kidney injury: a meta-analysis." *BMC nephrology* 19 (2018): 1-11. |
| 4 | Males are more likely to develop dialysis requiring AKI | Database search for large cohort of hospitalized patients (*Hospital Episode Statistics database*) | Neugarten, Joel, Ladan Golestaneh, and Nitin V. Kolhe. "Sex differences in acute kidney injury requiring dialysis." *BMC nephrology* 19 (2018): 1-7. |
| 5 | Progression of renal disease is slower in females than in males | Meta-analysis of multiple patient cohorts | Neugarten, Joel, and Ladan Golestaneh. "Influence of sex on the progression of chronic kidney disease." *Mayo Clinic Proceedings*. Vol. 94. No. 7. Elsevier, 2019. |
| 6 | Males are more likely to develop post-operative AKI than females of younger age. | Post-operative (non-cardiac, non-AKI surgery) patients | Privratsky, Jamie R., et al. "Postoperative acute kidney injury by age and sex: a retrospective cohort association study." *Anesthesiology* 138.2 (2023): 184. |
| 7 | Males are more susceptible to AKI, and renal-protective effect in females might be lost during aging. | Meta-analysis of multiple patient cohorts (*ADQI meeting*) | Soranno, Danielle E., et al. "The Role of Sex and Gender in Acute Kidney Injury-Consensus Statements from the 33rd Acute Disease Quality Initiative." *Kidney International* (2025). |
| 8 | Males have a higher risk of AKI than females of fertile age and aged-females under hormonal therapy | Prospective cohort of hospitalized patients | Golestaneh, Ladan, et al. "Sex, acute kidney injury, and age: a prospective cohort study." *American Journal of Kidney Diseases* 85.3 (2025): 329-338. |
